# Hybrid Predictive Machine Learning Model for the Prediction of Immunodominant Peptides of Respiratory Syncytial Virus

**DOI:** 10.3390/bioengineering11080791

**Published:** 2024-08-05

**Authors:** Syed Nisar Hussain Bukhari, Kingsley A. Ogudo

**Affiliations:** 1 National Institute of Electronics and Information Technology (NIELIT), Ministry of Electronics and Information Technology (MeitY), Government of India, Srinagar 191132, India; 2 Department of Electrical & Electronics Engineering, Faculty of Engineering and the Built Environment, University of Johannesburg, Johannesburg 0524, South Africa; kingsleyo@uj.ac.za

**Keywords:** respiratory syncytial virus, immunodominant peptides, T-cell epitope, peptide-based vaccine, hybrid, predictive model, machine learning

## Abstract

Respiratory syncytial virus (RSV) is a common respiratory pathogen that infects the human lungs and respiratory tract, often causing symptoms similar to the common cold. Vaccination is the most effective strategy for managing viral outbreaks. Currently, extensive efforts are focused on developing a vaccine for RSV. Traditional vaccine design typically involves using an attenuated form of the pathogen to elicit an immune response. In contrast, peptide-based vaccines (PBVs) aim to identify and chemically synthesize specific immunodominant peptides (IPs), known as T-cell epitopes (TCEs), to induce a targeted immune response. Despite their potential for enhancing vaccine safety and immunogenicity, PBVs have received comparatively less attention. Identifying IPs for PBV design through conventional wet-lab experiments is challenging, costly, and time-consuming. Machine learning (ML) techniques offer a promising alternative, accurately predicting TCEs and significantly reducing the time and cost of vaccine development. This study proposes the development and evaluation of eight hybrid ML predictive models created through the permutations and combinations of two classification methods, two feature weighting techniques, and two feature selection algorithms, all aimed at predicting the TCEs of RSV. The models were trained using the experimentally determined TCEs and non-TCE sequences acquired from the Bacterial and Viral Bioinformatics Resource Center (BV-BRC) repository. The hybrid model composed of the XGBoost (XGB) classifier, chi-squared (ChST) weighting technique, and backward search (BST) as the optimal feature selection algorithm (ChST−BST–XGB) was identified as the best model, achieving an accuracy, sensitivity, specificity, F1 score, AUC, precision, and MCC of 97.10%, 0.98, 0.97, 0.98, 0.99, 0.99, and 0.96, respectively. Additionally, K-fold cross-validation (KFCV) was performed to ensure the model’s reliability and an average accuracy of 97.21% was recorded for the ChST−BST–XGB model. The results indicate that the hybrid XGBoost model consistently outperforms other hybrid approaches. The epitopes predicted by the proposed model may serve as promising vaccine candidates for RSV, subject to in vitro and in vivo scientific assessments. This model can assist the scientific community in expediting the screening of active TCE candidates for RSV, ultimately saving time and resources in vaccine development.

## 1. Introduction

Respiratory syncytial virus (RSV) stands as a prominent contributor to lower respiratory tract ailments in both young children and the elderly [1]. The initial identification of RSV traces back to 1955, when it was first isolated from chimpanzees displaying respiratory symptoms at the Walter Reed Army Institute of Research in the United States [2]. Over subsequent years, the virus was also discovered in infants who suffered from severe lower respiratory illnesses [3,4]. Since that time, RSV has become known as a widespread pathogen, affecting almost every child by the age of two, with around half of them experiencing two infections within this period. [5]. The primary modes of transmission involve respiratory droplets released during coughs or sneezes, as well as direct contact with contaminated surfaces. Infants, young children, and older adults, particularly those with chronic medical conditions, face an elevated risk of severe illness due to RSV infection [6,7]. People infected with RSV are usually contagious for a period ranging from 3 to 8 days, with the potential to spread the virus a day or two before showing any symptoms [8]. However, some infants and individuals with weakened immune systems can remain contagious even after their symptoms have resolved, occasionally for as long as four weeks. [8]. The typical symptoms of an RSV infection include a runny nose, decreased appetite, coughing, sneezing, fever, and wheezing, which tend to develop gradually rather than all at once [9]. RSV is the most common cause of bronchiolitis and pneumonia in children under one year old [7]. The CDC estimates that RSV causes approximately 58,000 to 80,000 hospitalizations and 100 to 300 deaths among children under five annually. Additionally, it results in 60,000 to 160,000 hospitalizations and 6000 to 10,000 deaths each year among adults aged 65 and older [7]. RSV, which circulates during the winter months alongside influenza (flu) and other respiratory viruses, is frequently misdiagnosed due to its similar symptoms. Like the flu, its prevalence peaks between November and May [10].

**Figure 1:**
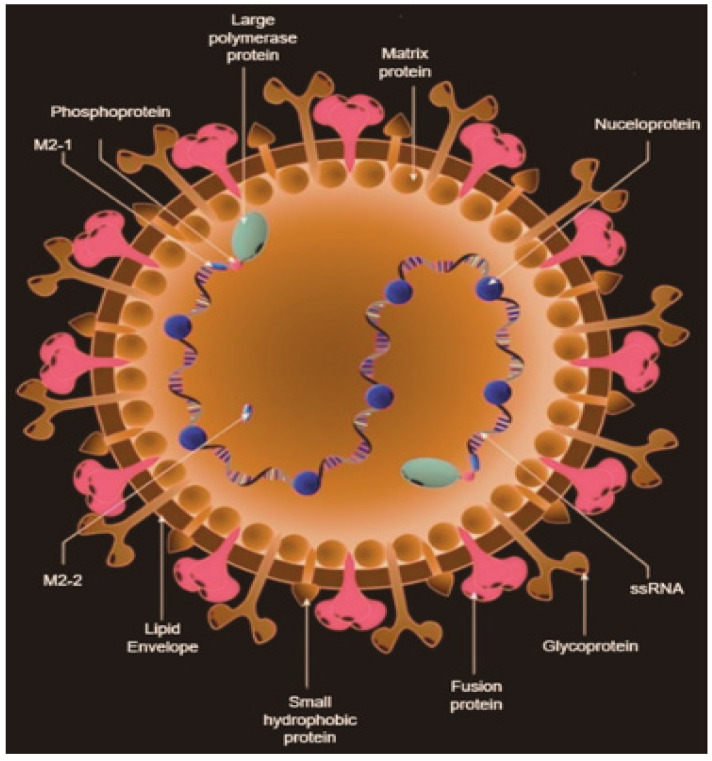
Structure of RSV.

RSV is categorized as a filamentous enveloped virus and is part of the *Orthopneumovirus* genus within the *Pneumoviridae* family, under the order *Mononegavirales* [1]. This virus is characterized by its genetic structure, which consists of a single-stranded RNA genome with a negative sense. This genome includes 11 proteins encoded by a 15.2-kilobase (kb) RSV genome. Unlike influenza, RSV possesses a non-segmented genome, which means it lacks the capacity to re-assort genome segments. As a result, RSV cannot undergo the genetic rearrangements known as antigenic shifts, which can lead to major pandemics [11]. RSV particles come in various shapes, including both spherical and filamentous forms of different sizes [12]. These virions have three surface proteins: F, G, and SH (small hydrophobic), as shown in Figure 1 [13]. The G and F proteins are crucial for virion attachment and fusion, binding to specific carbohydrate structures known as GAGs and RhoA, respectively [14,15]. Once fusion takes place, the virion releases its nucleocapsid into the cytosol, permitting the RNA to enter the host cell. The M2 mRNA contains two overlapping open reading frames (ORFs) that code for M2-1 and M2-2. The M2-2 gene regulates the shift from transcription to genomic RNA production [16]. The large (L) protein functions as a viral RNA-dependent RNA polymerase, encompassing multiple enzyme activities essential for RSV replication. This protein enters the genome, facilitating mRNA transcription. During replication, a complete positive-sense RNA complement of the genome called the antigenome is produced and serves as a template for further replication. Throughout this process, the N protein encapsulates the RNA, protecting it from degradation. The M protein plays a crucial role in coordinating the assembly of envelope proteins with nucleocapsid proteins (N, P, and M2-1). It also aids in the budding of new immature virions, a process that uses the host cell membrane. In filamentous virions, a helical arrangement of M (matrix) proteins is present, which is critical for forming infectious filamentous particles [17].

As shown in Figure 1, the RNA genome of RSV includes 10 genes that encode a total of 11 proteins. These proteins include two nonstructural proteins (NS1 and NS2). Additionally, there are four envelope proteins: the attachment glycoprotein (G), the fusion protein (F), the matrix protein (M), and the small hydrophobic protein (SH). Moreover, there are five ribonucleocapsid proteins: the nucleoprotein (N), phosphoprotein (P), large RNA polymerase (L), M2-1 (a transcription antiterminator that binds zinc), and M2-2 (a regulatory factor involved in balancing RNA replication and transcription) [18]. In vaccine development, particular focus is given to the F protein. This is due to its presence on the outer envelope of the RSV virion and its high conservation across different RSV strains, making it a promising target for vaccine development. The F protein exists in two forms, prefusion and postfusion, with the prefusion form being less stable but more immunodominant compared to the postfusion form [18].

To prevent disease outbreaks effectively, there is an urgent need to develop a safe and effective RSV vaccine that can induce immunological memory without causing immune-related complications following natural RSV infections [19]. Current vaccine development efforts have emphasized whole-organism vaccines, including live attenuated and inactivated types. However, these vaccines can be costly to produce, require the cultivation of the infectious agent, and may cause vaccine-related illnesses in recipients [20]. Additionally, they may not be suitable for individuals with compromised immune systems and require precise temperature control for storage [21]. As a result, there has been a shift towards developing peptide-based vaccines (PBVs). PBVs involve identifying and chemically synthesizing immunodominant peptides, known as T-cell epitopes (TCEs), which can elicit specific immune responses against the pathogen [22]. The design of PBVs focuses on removing unnecessary antigenic components, concentrating only on protein sections capable of triggering an immune response [23,24]. PBVs offer several advantages over traditional vaccines, including fewer side effects, simpler manufacturing processes, the absence of whole pathogen elements, increased specificity, greater stability, sustainability, and shorter production timelines [25]. Despite these significant advantages, PBVs have received less attention, and their potential to enhance vaccine safety and immunogenicity remains largely unexplored [26,27].

It is important to emphasize the vital role of T cells in adaptive immunity, as they aid in various immune system functions and significantly contribute to the control, clearance, and protection against most viral infections. [28]. Notably, CD8+ T cells are pivotal in the context of RSV pathogenesis, and there is a suggestion that RSV vaccines capable of inducing both antibodies and CD8+ T cells may prove effective [29]. The consideration of vaccines that trigger CD8+ T-cell responses against both cancer and viruses is a promising avenue in vaccine design [30,31], underscoring the idea that T cells are well equipped to address evolving viral variants [32,33]. Identifying these immunodominant TCEs for PBV design through wet-lab experiments is challenging, costly, and time-consuming. However, the application of machine learning (ML) techniques can enable the accurate prediction of these epitopes, expediting vaccine development and making it more cost-effective compared to traditional wet-lab methods [34]. This study presents a novel method for predicting the TCEs of RSV using a hybrid ML technique that leverages the physicochemical properties of peptides. The identified epitopes could be utilized as candidates in the development of PBVs against the RSV pathogen. The proposed model aims to aid the scientific community in identifying new and immunodominant TCEs specific to RSV.

### Contributions

This study makes several significant contributions. Firstly, it involved the development and testing of eight hybrid ML predictive models created through various permutations and combinations of two classification techniques, two feature weighting methods, and two feature selection strategies, all aimed at predicting the TCEs of RSV. Secondly, an innovative feature extraction technique was introduced, capable of extracting the physicochemical properties of peptides at the amino acid level. Thirdly, the study employed heuristic and greedy search techniques to identify optimal features for model training after extracting features from peptide sequences. Fourthly, the research primarily focused on achieving high accuracy in TCE prediction, and the proposed hybrid techniques demonstrated promising results in terms of accuracy. These models were thoroughly evaluated using multiple parameters, including area under the curve (AUC), sensitivity, specificity, Gini, F-score, and MCC. The findings indicate that the combination of XGBoost with chi-squared and backward search is the most accurate and reliable predictive method for TCE prediction in the context of RSV. Finally, K-fold cross-validation (KFCV) was performed, demonstrating that the proposed model is reliable and consistent for TCE predictions across all folds.

## 2. Related Work

Considerable research has been conducted to identify the TCEs of RSV for the design of PBVs. Chen et al. predicted T-cell epitopes in RSV F and G proteins, finding three RSV-A and two RSV-B clusters, indicating diverse immunogenic profiles. Recent epidemic strains conserved more F protein epitopes but reduced G protein epitopes. This study offers a framework for studying RSV T-cell epitope evolution, crucial for vaccine design [35]. The study [36] aimed to identify RSV-specific T-cell epitopes in BALB/c mice. Novel CD8 T-cell epitopes in the F and G proteins and previously unknown CD4 T-cell epitopes in P, L, M2-1, and N proteins were discovered. Longer 17-mer CD4-T-cell epitopes proved more effective in stimulating CD4-T-cell responses compared to 15-mer peptides. This work addresses the lack of defined RSV-specific T-cell epitopes, enhancing our understanding of RSV-induced disease. Another study [37] focused on designing a potential vaccine for RSV. Using reverse vaccinology, researchers predicted 95 cytotoxic T-lymphocyte (CTL) epitopes from the RSV proteome. After extensive screening for antigenicity, allergenicity, and toxicity, 70 epitopes with desirable properties were selected. Molecular docking identified stable binding in four epitopes, validating their potential as T-cell-specific RSV antigens. This approach provides an efficient method for screening immunogenic epitopes, offering promise for vaccine development against RSV. In [38], the authors aimed to identify CD4+ and CD8+ T-cell epitopes in C57BL/6 mice infected with RSV. Using an overlapping peptide library encompassing the RSV proteome, researchers discovered two new CD4+ and three new CD8+ T-cell epitopes within various RSV proteins. Additionally, they characterized these newly identified epitopes, including their TCR Vb expression profiles and MHC restriction. These findings will advance future research on RSV-specific T-cell responses in C57BL/6 mice. Shah et al. [39] focused on the potential use of epitope-based vaccines against RSV, which poses a significant threat to infants and the elderly. The study specifically targeted the fusion glycoprotein of RSV (RSV-FP) due to its conservation across strains and its ability to elicit cytotoxic T-cell (CTL) responses, crucial for viral clearance. Using immunoinformatics tools, the researchers identified seven 9-mer peptides within RSV-FP that strongly bind to 17 different HLA types, exhibit 100% sequence conservancy, and are estimated to provide a 76.03% population coverage worldwide. These findings hold promise for the development of effective RSV epitope-based vaccines. In this immunoinformatics study [40], researchers aimed to design a multi-epitope vaccine against RSV. They identified eight CD8-T-cell and three CD4-T-cell epitopes from glycoproteins F and G, considering antigenicity and binding affinity. Molecular docking confirmed strong associations with HLA alleles. Using these epitopes, a stable, non-allergenic, and antigenic multi-epitope vaccine with a cholera toxin-derived adjuvant was designed. Computational simulations indicated the vaccine’s potential to generate antibodies and effector T cells. Codon optimization and in silico cloning ensured enhanced expression in Escherichia coli. Further experimental validation is expected to confirm the vaccine’s effectiveness against RSV infections. A study [41] aimed to examine the role of vaccine-induced CD8+ T cells in protecting against RSV. Using a peptide vaccine (TriVax) in mice, researchers discovered that it induced strong anti-RSV CD8+ cytotoxic T lymphocytes. These vaccinated mice were protected against RSV infection, airway mucin expression, and lung inflammation when challenged six days post-vaccination. While effector CD8+ T cells exhibited strong cytokine expression and provided protection, memory CD8+ T cells, elicited 42 days post-vaccination, offered partial protection with lower cytokine expression, suggesting a link between protection and CD8+ T cell cytokine levels. Another study [42] aimed to develop a vaccine against RSV that induces long-lasting immunological memory without causing immunopathology. Researchers used live attenuated influenza vaccine (LAIV) viruses with RSV epitopes integrated into the neuraminidase or NS1 genes. These chimeric vaccines protected against both influenza and RSV without causing harmful effects. The study focused on CD4- and CD8-T-cell responses, particularly lung tissue-resident memory T-cell subsets (TRM). The RSV epitopes did not impact influenza-specific CD4 memory T cells, and both LAIV+NA/RSV and LAIV+NS/RSV vaccines induced strong RSV-specific CD8 TRM cells in the lungs. This research indicates that LAIV-based vaccines can generate robust localized T-cell immunity against foreign pathogens without compromising the vaccine’s immunogenicity. The authors of [43] reviewed computational tools for predicting T-cell epitopes, with a particular focus on neoepitopes relevant to cancer immunotherapy. They assessed various tools based on their methodologies, data utilization, and comparative advantages and disadvantages. The authors of [44] investigated the impact of antigen processing on epitope immunogenicity. They developed an ML model to predict proteasomal degradation scores for peptides and experimentally tested peptides with varying scores. Their findings suggest a correlation between low degradation scores and enhanced T-cell activation, highlighting the potential for improving vaccine efficacy by optimizing antigen processing. The study [45] addressed the challenge of epitope prediction for malaria due to the unique biology and evolving sequences of the parasite. The authors proposed an ML approach to develop a Plasmodium-specific epitope predictor. They built models using various ML algorithms trained on epitope data with sequence features and physicochemical properties. Their analysis suggests a model trained with specific classifiers after preprocessing outperforms others. This research represents the first in silico attempt to benchmark Plasmodium epitopes using ML and paves the way for peptide-based predictors in malaria vaccine development. The study [46] reviewed various in silico methods for predicting SARS-CoV-2 T-cell epitopes, highlighting the importance of T-cell responses in COVID-19. The authors compared various ML-based approaches by evaluating their ability to identify experimentally validated immunogenic epitopes. This review provides insights into the performance of different prediction methods and suggests future research directions.

## 3. Materials and Methods

**Figure 2:**
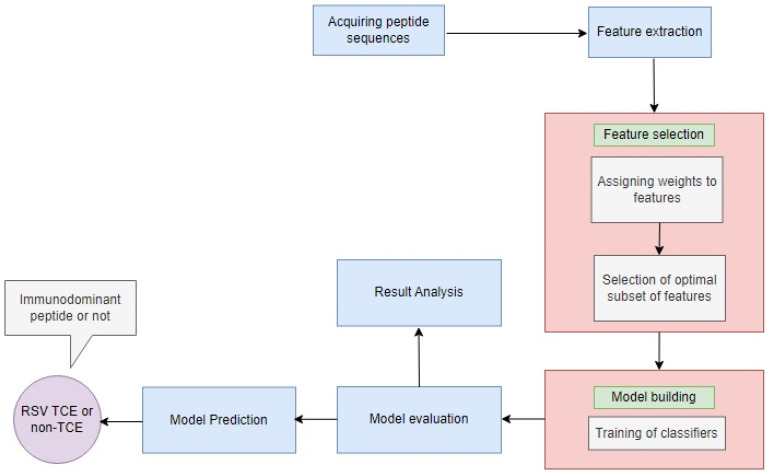
Proposed methodology.

In this section, as outlined in Figure 2, we will explain the proposed hybrid approach for predicting the IPs of RSV through the following sub-sections.

### 3.1. Retrieval of Peptide Sequences

The TCE and non-TCE (NTCE) peptide sequences were retrieved in the form of two CSV files (a TCE sequences file and a non-TCE (NTCE) sequences file) from publicly available repositories, namely, the “Bacterial and Viral Bioinformatics Resource Center (BV-BRC)” in CSV format [47,48]. To perform the binary classification, a target variable called “Class” was added to the CSV file. This variable has a value of 1 for TCE sequences and 0 for NTCE sequences.

### 3.2. Feature Extraction

The distinct characteristics of each peptide sequence are determined by the specific arrangement of amino acids and their related physicochemical properties, which are central to this study. To extract these properties from the peptide sequences outlined in Section 3.1, we carried out feature extraction (FE) using the peptides [49] and peptider [50] packages within the R programming environment [51]. Before performing FE, the duplicate peptide sequences were removed. The FE process produced a high-dimensional dataset formatted as a CSV file containing 108 features for each sequence. The details of the physicochemical properties used in this analysis are listed in Table 1, and Table 2 presents a snapshot of the dataset after FE.

### 3.3. Feature Selection

Feature selection (FS) is a crucial phase in the ML pipeline that focuses on identifying and choosing the most pertinent features from a dataset to develop a predictive model. The aim of FS is to minimize the number of features while preserving those that are most valuable for improving the model’s prediction accuracy [52]. This procedure not only aids in mitigating computational expenses and curbing overfitting but also enhances the model’s ability to generalize. The selected features should exhibit independence to avert redundancy and multicollinearity, factors that could compromise the model’s stability and comprehensibility. Due to the high dimensionality of the dataset, it is important to assign weights to all features and subsequently identify the optimal subset to improve the efficiency of the ML model. The following subsections offer an overview of the process for assigning feature weights and determining the optimal feature subset.

### 3.4. Assigning Weights to Features-Feature Weighting

The feature weighting technique (FWT) in ML involves assigning weights to features to control their influence on the model’s output. The goal of FWT is to increase the importance of relevant features while reducing the impact of irrelevant ones, thereby improving the model’s accuracy and robustness [53]. In this study, two different FWTs, namely the information gain technique (IGT) and the chi-squared technique (ChST) from the FSelector package in R were used to assign weights to the features [54]. A brief description of each technique is provided next.

#### 3.4.1. IGT

Information gain (IG) measures the amount of information obtained when a feature is used to split the data. In other words, it measures the reduction in entropy or uncertainty about a random variable after observing another random variable. In the context of feature selection, IG evaluates how well a feature separates the training examples according to their target class. Higher values indicate that a feature is more effective in distinguishing between classes. The function prototype for IG is represented as information.gain(x, y, …), where x and y are the required parameters corresponding to the feature and class variables, respectively. The formula for information gain is as follows:

IG(T,A) = H(T) − H(T∣A)

where

- IG(T,A) is the information gain of feature A for target T.
- H(T) is the entropy of the target variable T.
- H(T∣A) is the conditional entropy of T given feature A.

#### 3.4.2. ChST

The chi-squared technique evaluates the independence between a feature and the class variable by measuring the difference between observed and expected frequencies of each class across the feature’s categories. In other words, the ChST test is used to determine whether there is a significant association between a categorical feature and the target variable. It measures the discrepancy between the observed and expected frequencies of occurrences. Higher values indicate a stronger association between the feature and the class variable. The function chi.squared(x, y, …) accepts the same parameters as the IGT. The formula for the chi-squared statistic is as follows:

χ2 = ∑ (O_i_ − E_i_)^2^/E_i_

where

- O_i_ is the observed frequency for category i.
- E_i_ is the expected frequency for category i, assuming no association between the feature and the target.

### 3.5. Selection of Optimal Subset of Features

Once weights are assigned to the features, identifying the optimal feature subset becomes imperative for constructing a precise and efficient ML model. The optimal feature subset (OFSS) plays a pivotal role in achieving optimal predictive performance [55]. Selecting the OFSS involves choosing a subset of features from a larger set that provides the most accurate predictions for the ML model. This step is especially important in high-dimensional datasets, where an excess of features can negatively affect model performance if not properly managed. To identify the optimal feature subset, two effective techniques are used in this study: the hill climbing search technique (HCST) and the backward search technique (BST). A brief overview of each OFSS technique employed in this study is provided next.

#### 3.5.1. HCST

The HCST is a heuristic optimization method that systematically evaluates the performance of different feature subsets, ultimately selecting the one that achieves the highest accuracy. Starting with an initial subset of features, HCST explores the performance of all possible one-feature additions and identifies the subset that offers the most significant improvement in accuracy [56]. This process continues iteratively until no further enhancements in accuracy can be achieved.

#### 3.5.2. BST

The BST begins with the full set of features and methodically removes those that contribute least to improving accuracy. BST evaluates the impact of removing each feature and selects the subset that offers the greatest increase in accuracy [57]. This iterative process continues until no further gains in accuracy can be achieved.

### 3.6. Selection of ML Classifiers 

The final hybrid techniques were developed by combining two widely used classification methods: XGBoost (extreme gradient boosting) and random forest (RF). The results from the aforementioned optimal feature selection techniques were used as inputs for these two classification methods. This strategy was employed in the final classification phase to allow for comparative analysis, as each method utilizes different approaches to classify the data. Typically, random forest (RF) consists of an ensemble of decision trees, where multiple trees are built, and the final classification is determined by majority voting [58]. On the other hand, XGBoost is an ML algorithm renowned for its efficiency and effectiveness in classification tasks [59]. It operates by iteratively adding decision trees to minimize the residual errors from previous trees, thereby optimizing a specified loss function to produce a highly accurate ensemble model. Table 3 outlines these ML classifiers alongside their respective tuning parameters.

### 3.7. Model Building 

The proposed models were constructed by combining two FWTs, two OFSS techniques, and two classification methods using permutation and combination approaches. Initially, the peptide sequences of RSV were obtained from BV-BRC in CSV format. Feature extraction (FE) was then performed using the “peptides” and “peptider” packages in the R programming language, resulting in high-dimensional data with 108 features extracted for each peptide sequence. After the FE process, the next step was to assign weights to each feature to assess its relative importance. This was accomplished using two different FWTs. Following this, the OFSS was determined using two distinct techniques. Each FWT output was fed into each OFSS technique separately. The results from each OFSS were then used to train two different classification algorithms. The optimal features identified by various FS methods are listed in Table 4. Equations (1) and (2) illustrate the model formulas with the dependent variable “Class” and its corresponding independent variables for training via the hill climbing and backward search techniques, respectively.

Class∼f (F4, F7_18,…………, F8_37, F10)(1)

Class∼f (F2, F5_2,……………, F9_6, F9_8)(2)

Next, the dataset with the optimal features from each combination of FWTs and OFSS techniques was divided into training and testing sets. The data were split at a ratio of 70:30, with 70% allocated for training the model and the remaining 30% reserved for testing. After training, the models were validated using the evaluation metrics described in the following section. To ensure the models’ robustness and reliability, K-fold cross-validation was performed.

## 4. Model Evaluation

Model evaluation constitutes a crucial aspect of any machine learning (ML) workflow. It is imperative to assess the performance of the ML model to ensure its capability to generalize accurately to new, unseen data [60]. This approach aids in the identification of the optimal model that effectively represents the data and predicts future performance. In this study, a variety of assessment metrics were used, including accuracy, sensitivity, specificity, precision, area under the receiver operating characteristic curve (AUROC), F1 score, and Matthews correlation coefficient (MCC) [61]. Additionally, the performance consistency and robustness of the proposed techniques were evaluated using K-fold cross-validation (KFCV). It is important to note that evaluating ML models is an iterative process. The outcomes of these evaluations can inform adjustments to the model, including adjustments to hyperparameters, feature selection, or data preprocessing. This iterative process continues until the model reaches the desired level of performance. The following section details the metrics used for model evaluation, where TP stands for true positive, TN for true negative, FP for false positive, and FN for false negative.

### 4.1. Accuracy

The accuracy metric evaluates the proportion of correct predictions made by the model relative to the total number of predictions [62]. It is computed using Equation (3).

Accuracy = (TP + TN)/(TP + TN + FP + FN)(3)

### 4.2. Sensitivity

Sensitivity, also referred to as recall or true positive rate (TPR), is a metric used to evaluate ML models by measuring the proportion of actual positives that are correctly identified by the model [63]. Recall is calculated using Equation (4).

Sensitivity = TP/(TP + FN)(4)

### 4.3. Specificity

Specificity, or true negative rate (TNR), is a metric that gauges the proportion of actual negatives that are accurately identified by the model [63]. Specificity is calculated using Equation (5).

Specificity = TN/(TN + FP)(5)

### 4.4. Precision

Precision, also called positive predictive value (PPV), is a metric that assesses the proportion of positive predictions that are genuinely correct [63]. Precision is calculated using Equation (6).

Precision = TP/(TP + FP)(6)

### 4.5. F1 Score

The F1 score is a metric used to evaluate ML models by combining both precision and recall into a single measure of performance [63]. It represents the harmonic mean of precision and recall and is calculated using Equation (7).

F1 score = 2 × (precision × recall)/(precision + recall)(7)

The F1 score is represented as a value between 0 and 1, with higher values indicating better model performance in accurately identifying both positive and negative classes.

### 4.6. Area under the ROC Curve (AUC-ROC)

The receiver operating characteristic (ROC) curve plots the true positive rate (TPR) against the false positive rate (FPR) for various threshold settings [64]. The area under the ROC curve (AUC-ROC) measures the model’s overall ability to differentiate between positive and negative classes. It is a single metric ranging from 0 to 1, where a value of 1 denotes a perfect classifier, and a value of 0.5 represents a random classifier.

### 4.7. Mathews Correlation Coefficient (MCC)

The Matthews correlation coefficient (MCC) takes into account all four values from the confusion matrix (TP, FP, TN, FN) and provides a score between −1 and 1. A score of 1 represents a perfect prediction, 0 indicates a random prediction, and −1 denotes a prediction that is completely opposite to the true labels [65]. The MCC is calculated using Equation (8).

MCC = (TP × TN − FP × FN)/(Sqrt ((TP + FP) × (TP + FN) × (TN + FP) × (TN + FN)))(8)

### 4.8. K-Fold Cross Validation (KFCV)

**Figure 3:**
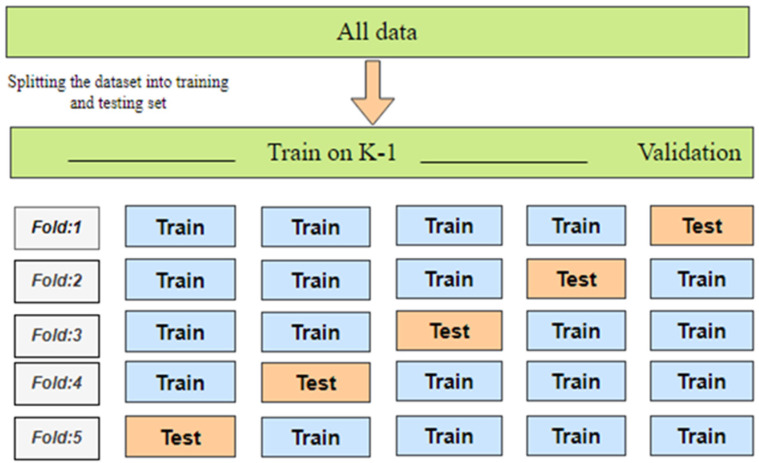
K-fold cross-validation technique.

K-fold cross-validation (KFCV) is a method used to evaluate the consistency and robustness of a model by dividing the original dataset into K subsets, or folds, of approximately equal size [66]. The model is trained and tested K times, with each fold serving as the evaluation set once while the remaining K-1 folds are used for training, as illustrated in Figure 3. The typical KFCV process includes several steps: First, the dataset is randomly shuffled to ensure even distribution. Next, the dataset is split into K groups or folds of roughly equal size. Then, the model is trained on K-1 folds and tested on the remaining fold for each iteration. Performance metrics, such as accuracy, are computed for each fold. Finally, the mean and variance of these metrics are calculated across the K folds to provide an overall assessment of the model’s performance. KFCV helps reduce the risk of overfitting and offers a more reliable estimate of the model’s effectiveness on new data. The value of K is usually set to 5 or 10, though it can be adjusted based on the dataset size and model complexity.

## 5. Results and Discussion

In this section, we present the results obtained from applying various hybrid techniques to a high-dimensional dataset, which comprises 108 features extracted from RSV peptide sequences. To determine the most effective hybrid technique, a comparative analysis was performed among the different hybrid methods used in this study, based on the evaluation parameters previously outlined. Table 5 presents the accuracies achieved by the various hybrid approaches. It is evident from Table 5 that XGBoost (XGB) demonstrates outstanding results, consistently exceeding 93% accuracy across all scenarios.

Notably, the hybrid approach combining ChST and BST achieves the highest accuracy of 97.29% for XGBoost (XGB) models. In terms of accuracy, random forest (RF) with various feature weighting (FW) and optimal feature selection techniques shows a range of accuracy from a low of 79.23% to a high of 94.19% with IGT and HCST among the different hybrid techniques used in this study. However, when evaluating the effectiveness of a hybrid model in a multiclass problem, accuracy alone does not suffice as the sole determining factor [60]. Other crucial parameters such as recall, specificity, precision, negative predicted value of a particular class, AUROC, and F1 score of the predictive method must also be considered. To this end, Table 6 presents a comprehensive comparison of these parameters for the best hybrid models achieved in this study. As depicted in Table 6, the XGB model (Model 1) in combination with the chi-squared and backward search techniques demonstrates superior results across all parameters, boasting an impressive F1 score and AUROC value of 0.98 and 0.99, respectively.

**Figure 4:**
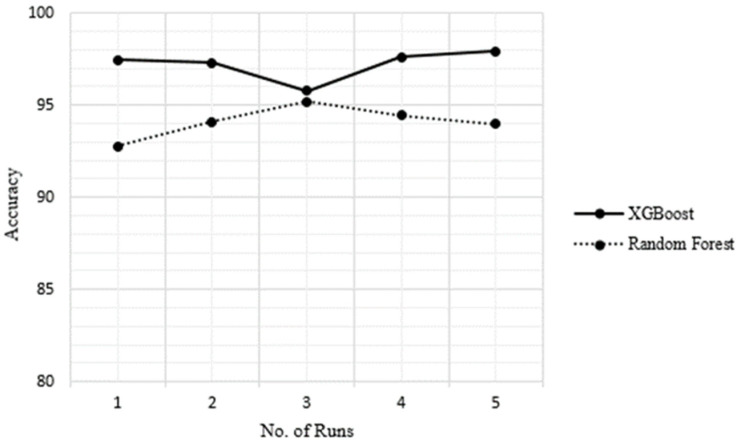
KFCV results of the hybrid model as depicted in Figure 4; it is evident that the hybrid XGBoost model exhibits the most consistent accuracy results compared to the RF hybrid techniques. The results indicate that the proposed model stands out due to its comprehensive hybrid framework that combines multiple feature weighting, selection, and classification techniques, aiming to capture diverse peptide characteristics for improved accuracy. Unlike many existing tools relying on single models or limited feature engineering, the proposed approach leverages the strengths of different algorithms to mitigate potential biases. Moreover, the KFCV technique mitigates the risk of overfitting by dividing the dataset into K equal-sized folds. The model is trained on K-1 folds and tested on the remaining fold iteratively. This process provides a more reliable estimate of model performance on unseen data by exposing the model to different subsets of the data during training.

Assessing the reliability of the technique is essential to determine whether the model is susceptible to overfitting or underfitting issues. Overfitting occurs when the model excels with training data but fails to generalize to testing data, while underfitting happens when the model performs poorly on both training and testing data. To verify the reliability and consistency of the hybrid techniques used in this study, 5-fold cross-validation (5 FCV) was performed on the top three hybrid methods. The accuracies achieved by these top-performing hybrid models across different folds are shown in Table 7, and their accuracy is plotted in Figure 4.

## 6. Conclusions

In conclusion, RSV poses a significant threat to individuals across all age groups, especially infants and young children, with seasonal outbreaks typically peaking during autumn and winter months. Vaccination remains the most effective strategy for managing viral disease outbreaks [67]. While ongoing efforts aim to develop an RSV vaccine, many current methods involve using weakened forms of the entire pathogen to trigger an immune response. In contrast, the potential B-cell vaccine (PBV) concept emphasizes the identification and synthetic creation of specific immunodominant peptides, known as T-cell epitopes (TCEs), as potential components of a vaccine. Despite the many advantages of PBVs, such as enhanced safety, immunogenicity, and cost-effectiveness, they have not received widespread attention [68]. Computational methods provide a quicker and more economical way to identify TCEs compared to traditional laboratory techniques. In this study, we developed and assessed eight hybrid predictive ML models for forecasting the TCEs of RSV [69]. After extracting features from peptide sequences, we used heuristic and greedy search techniques to identify the most effective features for model training. Performance evaluation using various metrics, including accuracy, sensitivity, specificity, and AUROC curve, showed that the combination of XGBoost with ChST and BST was the most accurate and reliable predictive method. Our model provides deterministic TCE prediction, unlike other methods, such as NetMHC [70] and CTLpred [71], which only estimate binding potential. Furthermore, our model can predict peptides of various lengths, including those longer than 9-mers, addressing a limitation of CTLpred. However, it is crucial to validate model predictions through experimental methods (in vivo and in vitro) before considering them for vaccine development [72]. In summary, the hybrid ML techniques proposed in this study demonstrated exceptional performance and surpassed current ML methods for predicting RSV TCEs. Future research should explore additional physicochemical properties and utilize advanced ML classifiers to further improve accuracy and other metrics. Overall, using computational methods to identify potential vaccine candidates could significantly impact global health by saving lives, preventing future outbreaks, and reducing the virus’s capacity to evade immunity through genetic mutations.

## Figures and Tables

**Table 1:** Physicochemical properties.

Physicochemical Property	Count	Notation
Aliphatic index	1	F1
Boman index	1	F2
Insta index	1	F3
Probability of detection	1	F4
Hmoment index	2	F5_1, F5_2
Molecular weight	2	F6_1, F6_2
Peptide charge for 45 scales	45	F7_1 to F7_45
Hydrophobicity at 44 scales	44	F8_1 to F8_44
Isoelectric point for 9 pKscale	9	F9_1 to F9_9
Kidera factors	1	F10
aaComp	1	F11

**Table 2:** Snapshot of the dataset.

Peptide Sequence	F1	F2	-----	F10	F11	Class
VRSKVF	28.10	0.976	-----	−2.765	4.101	1
SRISKDAT	34.76	0.409	-----	−1.98	−1.342	1
KFELRZFIG	132.60	2.157	-----	−0.577	−0.029	0
SAVFEKTLS	97	−6.98	-----	−0.912	−4.719	0

**Table 3:** ML classifiers used.

Classifier	Method	Package	Tuning Parameter
XGB	xgb	xgboost	(booster = “gbtree”, objective = “binary:logistic”, max_depth = 6, min_child_weight = 1, subsample = 1)
RF	randomForest	randomForest	Ntree = 1500, mtry = 10

**Table 4:** Optimal feature sets by different FS techniques.

Technique	Optimal Feature Set	No. of Features
HcS	F7_43, F7_42, F1, F8_43, F7_38, F11_7, F7_28, F8_24, F4, F7_41, F8_13, F7_6, F7_22, F10, F8_23, F7_7, F7_19, F8_16, F9_2, F7_23, F5_2, F11_3	22
BST	F6_1, F9_4, F9_7, F7_8, F8_7, F7_37, F7_1, F5_2, F8_10, F3, F7_40, F7_39, F7_24, F8_19, F7_5, F1, F7_33, F8_20, F7_34, F7_38, F2, F9_5, F7_14, F11_13, F6_2	25

**Table 5:** Accuracies achieved by different hybrid models.

FWT	OFSS		CT
	BST	HCST	
IGT	93.65	95.12	
ChST	97.10	95.64	XGB
IGT	79.23	94.19	
ChST	91.32	93.68	RF

**Table 6:** Comparative results of best hybrid models.

Model	Sensitivity	Specificity	F1 Score	AUC	Precision	MCC
Model 1: ChST−BST–XGB	0.98	0.97	0.98	0.99	0.99	0.96
Model 2: IGT−HCST–RF	0.92	0.93	0.90	0.96	0.92	0.94

**Table 7:** Top two hybrid models’ accuracy via 5FCV.

Run	Model 1	Model 2
1	97.45	92.76
2	97.31	94.11
3	95.78	95.19
4	97.62	94.43
5	97.92	93.96
Average Accuracy	97.216	94.09

## Data Availability

The data that support the findings of this study are available on request from the corresponding author.
